# A Unique Case of Moraxella catarrhalis Meningitis Following Neurosurgical Intervention

**DOI:** 10.7759/cureus.59689

**Published:** 2024-05-05

**Authors:** Hussein Harb, Hasan Al-Obaidi, Kyvan Irannejad, Farshad Bagheri

**Affiliations:** 1 Basic Sciences, Ross University School of Medicine, Bridgetown, BRB; 2 Internal Medicine, Jamaica Hospital Medical Center, New York, USA; 3 Infectious Diseases, Jamaica Hospital Medical Center, New York, USA

**Keywords:** neurological diagnostic challenges, atypical bacterial meningitis, postoperative meningitis, neurosurgical infection, moraxella catarrhalis meningitis

## Abstract

We present a rare case of *Moraxella catarrhalis* meningitis in a 51-year-old immunocompetent woman after surgical resection of a fourth ventricle ganglioma. Notably, the patient had no history of sinusitis or otitis media, which are typical predisposing factors for *Moraxella* infection. She developed symptoms including headache, altered mental status, and neurological deficits three days post discharge, leading to her diagnosis confirmed by cerebrospinal fluid culture. This case highlights the diagnostic challenges and management complexities of atypical meningitis post neurosurgery. The occurrence emphasizes the necessity of considering *Moraxella catarrhalis* in differential diagnoses, particularly following neurosurgical procedures. This instance contributes to the scarce documentation of such infections in immunocompetent adults, underscoring the importance of vigilant microbiological evaluation and tailored antimicrobial therapy in postoperative settings.

## Introduction

*Moraxella catarrhalis*, once considered a harmless commensal of the upper respiratory tract, has become increasingly recognized as a significant pathogen, especially in susceptible populations, such as neonates, immunocompromised individuals, and those with chronic respiratory diseases [1]. Though often implicated with sinusitis and otitis media, this organism has also been documented to cause severe invasive infections like pneumonia, septicemia, and, though rare, meningitis [2]. This case of meningitis caused by *Moraxella catarrhalis* following neurosurgical procedures remains exceedingly rare, with few adult cases documented globally [3].

## Case presentation

A 51-year-old female patient with a medical history notable for constipation, hypertension, hyperlipidemia, prediabetes, and a cerebral infarct (date unspecified) presented with symptoms of slurred speech, right-sided weakness, and headache. The absence of sinusitis or otitis media in the patient's medical background was noted. Initial diagnostic imaging via a head CT scan identified a fourth ventricle ganglioma accompanied by hydrocephalus. To alleviate the immediate symptoms, an external ventricular drain (EVD) was promptly placed, followed by a surgical occipital craniotomy for tumor resection four days thereafter. Subsequent to the procedure, the EVD was removed after 10 days, and the patient was discharged in stable condition two weeks post operation, without any reported complications.

Three days post discharge, the patient developed symptoms including headache, altered mental status, slurred speech, and paresis of the right upper limb with decreased motor strength. Although she was hemodynamically stable, alert, and oriented, she was somnolent and responsive to verbal cues, scoring 10 on the Glasgow Coma Scale. Her neurological examination revealed significant right upper limb paresis, with motor strength of 4/5 in the deltoid, triceps, and wrist flexion/extension muscles, and 0/5 in the interossei muscles; these findings deviated from her baseline, which had none of these symptoms. Her exam was negative for meningeal signs, and the fundus was not examined. Additionally, the examination showed an elevated leukocyte count, xanthochromic cerebrospinal fluid (CSF), and a slight progression of previously noted hydrocephalus on a head CT scan. These developments led to her admission to the neurological critical care unit.

The patient was commenced on a therapeutic regimen consisting of ceftriaxone, ampicillin, vancomycin, acyclovir, and dexamethasone (RAVAD). Ceftriaxone was substituted with cefepime to address pseudomonal coverage due to recent EVD procedures. Following consultation with infectious disease (ID) specialists, vancomycin was discontinued upon gram-negative rod growth in the CSF culture. Ampicillin and dexamethasone were also discontinued based on the advice of the ID team. A repeat lumbar puncture was advised after four to five days of treatment, with potential consideration of intrathecal gentamicin if the CSF culture continued to yield gram-negative rods. Acyclovir therapy was sustained until negative results for herpes simplex virus (HSV) polymerase chain reaction.

**Figure 1:**
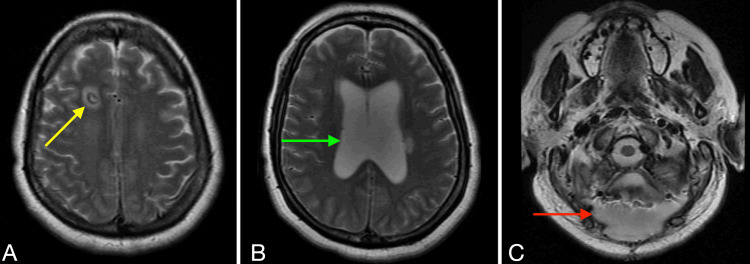
Axial 2D T2-weighted brain MRI analysis. (A) Frontal extraventricular drain: The image shows moderate enhancement along the drain tract, indicated by the tip of the yellow arrow. (B) Hydrocephalus: Moderate hydrocephalus is visible, located at the tip of the green arrow. (C) Suboccipital craniotomy postoperative changes: The image displays postoperative changes within the fourth ventricle, showing an accumulation of fluid external to the cranial vault, which indicates CSF leakage. This is found at the tip of the red arrow.

Further workup showed neutrophilic leukocytosis and hyponatremia, for which she received daily boluses of 3% hypertonic saline and salt tablets. Testing revealed high urine osmolality. MRI imaging with and without contrast showed no significant change from the time of discharge, as shown in Figure 1. Blood cultures showed no growth after five days. Final CSF cultures identified *Moraxella catarrhalis*. Our working diagnosis was altered mental status and right hemiparesis secondary to meningitis with the syndrome of inappropriate antidiuretic hormone secretion (SIADH) causing electrolyte imbalances.

Despite initial improvement in speech and upper extremity strength, the patient developed a mild headache, exacerbated by head turning but relieved by Tylenol. Physical examination revealed moderate head swelling at the surgical site and light yellow drainage, suggestive of a pseudomeningocele with CSF leakage. A lumbar drain was placed and the superior portion of the suboccipital craniotomy (SOC) was reapproximated to prevent further CSF leak. Cefepime was continued for antibiotic treatment.

**Figure 2:**
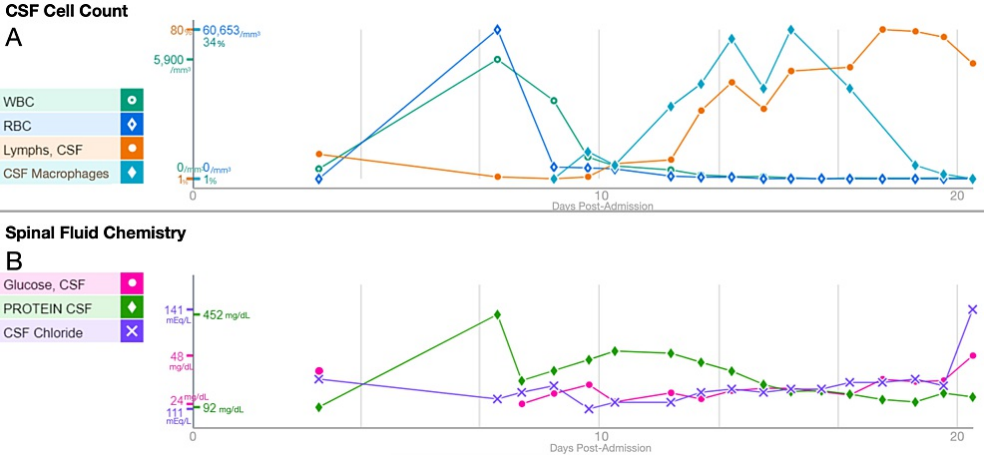
Analysis of cerebrospinal fluid (CSF) during hospitalization. (A) CSF cell count: This line graph displays changes in various cell types within the CSF over the course of the patient's hospital stay. It shows an initial rise followed by a decline in white blood cell (WBC) count per cubic millimeter, a biphasic pattern in red blood cell (RBC) count per cubic millimeter, a steady increase in the percentage of CSF lymphocytes (lymph), and a peak followed by a decrease in the percentage of CSF macrophages. (B) Spinal fluid chemistry: This line graph presents the biochemical changes in the CSF. It illustrates an overall increase in glucose levels (measured in mg/dL), an initial increase and subsequent decrease in protein levels (measured in mg/dL), and a rise in chloride levels (measured in mEq/L) following an initial decrease.

The patient's course was further complicated by spiking fevers and persistent signs of meningitis, as evidenced by cloudy, red, xanthochromic CSF with elevated white blood cell, protein levels, red blood cells, and low glucose, indicative of ongoing inflammation. ID recommended adding intravenous vancomycin and metronidazole to broaden the antimicrobial coverage. Following these adjustments, CSF and blood cultures remained negative, and a gradual improvement in CSF parameters was observed (Figure 2). In addition, the patient’s electrolyte imbalances were resolved. After completing a three-week course of cefepime and a 10-day course of metronidazole and vancomycin, the patient was started on a 14-day course of IV ceftriaxone due to its excellent coverage of *Moraxella catarrhalis*. During the ceftriaxone course, her symptoms of headaches and nuchal rigidity had completely resolved.

## Discussion

*Moraxella catarrhalis*, formerly known as *Neisseria catarrhalis*, is a gram-negative diplococcus primarily known as a commensal organism of the upper respiratory tract [4]. Historically underestimated, its significance as a pathogen has been recognized over recent decades, particularly in predisposing children, immunocompromised adults, and the elderly to respiratory infections, and increasingly in lower respiratory tract infections among adults with chronic conditions such as chronic obstructive pulmonary disease (COPD) [3-5]. Notably, its capability to cause severe infections like pneumonia, endocarditis, septicemia, and meningitis in immunocompromised individuals underscores its pathogenic potential [2].

The reported case presents a rare instance of *Moraxella catarrhalis* causing meningitis in an adult post-neurosurgical intervention, a context in which it is seldom documented. The likely pathogenesis in our case involves the perioperative acquisition, possibly during surgery or from nosocomial sources, given the absence of typical predisposing conditions like otitis media or sinusitis in our patient. Unlike most postoperative neurosurgical infections, which are typically caused by organisms like *Klebsiella* and *Staphylococcus aureus*, this patient was infected with *Moraxella catarrhalis* [6]. The organism's ability to invade and persist within the human respiratory tract, and potentially evade immune detection through mechanisms like complement inactivation, may contribute to its pathogenicity in vulnerable settings such as post-surgical interventions [7].

This case illustrates an adult presentation of *Moraxella catarrhalis* meningitis, which is exceptionally rare with fewer than 40 reported cases since the early 20th century [8]. The diagnosis was confirmed by CSF culture, which is critical given the bacterium's fastidious nature. The clinical features observed were typical of pyogenic meningitis, including CSF turbidity, leukocytosis with a predominance of polymorphonuclear cells, and elevated protein levels, coupled with decreased glucose levels.

The management of this case was complicated by the initial suboptimal response to broad-spectrum antibiotics, necessitating adjustments based on clinical progression. The presence of beta-lactamase-producing strains with 95% prevalence complicates empirical therapy, though most strains remain sensitive to a wide range of antibiotics [9]. This underlines the importance of tailored antibiotic therapy based on culture results, particularly in managing infections where *Moraxella* is a less common pathogen.

While vaccination against common pathogens like *Streptococcus pneumoniae* has altered the landscape of microbial colonization, increasing the prevalence of *Moraxella catarrhalis*, no vaccine is currently available for *Moraxella* due to challenges in establishing effective animal models and correlates of protection [10]. The economic impact of developing such a vaccine could be significant, considering the billions of dollars in healthcare costs associated with managing *Moraxella*-related infections such as otitis media and COPD-related infections [11].

The prognosis in *Moraxella catarrhalis* infections can be favorable with appropriate antibiotic therapy, as evidenced in pediatric cases [12,13]; however, adult cases, particularly those involving meningitis or other severe manifestations, require careful management and may have a more guarded outlook due to the potential for severe outcomes and complications.

## Conclusions

This unique case of *Moraxella catarrhalis* meningitis following neurosurgical intervention highlights several key aspects of this pathogen's evolving clinical significance. It underscores the necessity for heightened awareness among clinicians about atypical pathogens in atypical presentations, especially in patients with recent surgical interventions or other healthcare-associated risk factors.
